# The association of higher thyroid stimulating hormone levels in the normal range with unexplained infertility: A cross-sectional study

**DOI:** 10.18502/ijrm.v22i5.16434

**Published:** 2024-07-08

**Authors:** Mahdiehsadat Jalili, Reyhaneh Azizi, Abbas Aflatoonian, Akram Ghadiri-Anari

**Affiliations:** ^1^Diabetes Research Center, Shahid Sadoughi University of Medical Sciences, Yazd, Iran.; ^2^Research and Clinical Center for Infertility, Yazd Reproductive Sciences Institute, Shahid Sadoughi University of Medical Sciences, Yazd, Iran.

**Keywords:** Thyrotropin, Infertility, Thyroid hormones, Women.

## Abstract

**Background:**

Since thyroid hormones have an essential role in energy production, early development of the human placenta, embryo development, and implantation, abnormalities in thyroid function can significantly affect pregnancy outcomes.

**Objective:**

The present study aimed to investigate the effect of higher thyroid-stimulating hormone (TSH) levels in the normal range in euthyroid women with unexplained infertility.

**Materials and Methods:**

In this cross-sectional study, we evaluated the data for 300 euthyroid women aged between 18 and 39 yr with normal TSH levels ( ≤ 5 mIU/L) referred to Yazd Reproductive Sciences Institute, Yazd, Iran from December 2018-March 2021 in 2 groups: the case group (with unexplained infertility) and the control group (with male factor infertility). Finally, age, body mass index, and serum levels of TSH were extracted from participants' medical records and compared between groups.

**Results:**

The mean age and TSH level of participants were 31.52 ± 3.52 yr and 1.24 ± 2.59 mIU/L, respectively. 142 women (47.3%) had TSH < 2.5, and 158 women (52.7%) had TSH ≥ 2.5 mIU/L. The women with unexplained infertility had significantly higher TSH levels than controls in the same normal range (0.62 vs. 0.64 mIU/L, p < 0.001). Although a more significant proportion of women in the case group had TSH levels > 2.5 mIU/L, we did not find a significant association between TSH levels and age or body mass index.

**Conclusion:**

Women with unexplained infertility have a higher level of serum TSH in the normal range ( ≥ 2.5 mIU/L) than the control group. So, the effect of TSH treatment on these women should be considered.

## 1. Introduction

Infertile couples cannot achieve spontaneous pregnancy after at least 12 consecutive months of unprotected intercourse (1), which is a significant global health issue (2). According to the World Health Organization reports, 10–15% of young couples worldwide suffer from infertility, of which 8–27% is due to female infertility (2, 3). Unexplained infertility is diagnosed if infertility occurs in the following conditions: regular ovulation, normal fallopian tubes, normal uterine cavity, and normal semen analysis. Various factors, such as hyperprolactinemia as well as thyroid dysfunction, can lead to infertility (1). Since thyroid hormones have an important role in energy production, early development of the human placenta, embryo development, and implantation (1, 4), dysfunction and changes in thyroid hormone levels can significantly affect pregnancy outcomes (5).

Thyroid-stimulating hormone (TSH) levels are reportedly higher in women with unexplained infertility (6). According to the American Thyroid Association and the American Society of Reproductive Medicine, TSH levels should be considered in infertile women (1, 4).

Determining hormone factors essential to women's infertility may lead to effective and cost-effective treatment for couples and improve pregnancy rates. The answer to whether higher TSH levels in the normal range are associated with unexplained infertility is unknown. This study aimed to evaluate the effect of higher TSH levels in the normal range in women with unexplained infertility in Yazd, Iran.

## 2. Materials and Methods

### Participants

In this cross-sectional study, medical data of 300 infertile women aged between 18 and 39 yr who referred to Yazd Reproductive Sciences Institute, Yazd, Iran from December 2018-March 2021 were evaluated in 2 groups: the case group (with unexplained infertility) and the control group (with male factor infertility). Our inclusion criteria included infertile women aged between 18 and 39 yr with normal TSH levels ( ≤ 5 mIU/L), having normal menstrual cycles (21–35 days), normal uterus and fallopian tube in hysterosalpingography or laparoscopy, follicle-stimulating hormone level ≤ 10 IU/MI, and estradiol level ≤ 80 pg/Ml. Also, normal semen analysis (sperm concentration ≥ 15 million/ml, motility ≥ 40% and normal morphology ≥ 4%) in the case groups and the sperm concentration less than 1 million/ml, severe azoospermia, or oligospermia in the control group were considered as the inclusion criteria. All women with hypo- or hyperthyroidism, body mass index (BMI) < 18.5 or > 30 kg/m^2^, history of previous ovarian surgery, cervical stenosis, endometriosis, multiple ovarian cysts, endocrine disorders such as polycystic ovary syndrome, abortion, TSH levels > 5 mUI/ml, hyperprolactinemia, and incomplete medical record information were excluded.

### Sample size 

The sample size was estimated to be 150 in each group by considering the significance level of 95%, the power of 80%, 26% frequency for TSH = 2.5–5 for the case group and 12% for TSH < 5 for the control group, based on a previous study (1). 


$$n=\frac{{(Z_{1-\frac{a}{z}}+Z_{1-\beta })}^{2}\times (p_{1}\times \left(1-p_{1}\right)+p_{2}\times \left(1-p_{2}\right)}{{(p_{1}-p_{2})}^{2}}$$

### Study methods

Using the simple sampling method and according to inclusion and exclusion criteria, a total of 300 women were included in the study in group of 2, case and control (n = 150/each). Variables, including age, BMI, and serum levels of TSH, were extracted from participants' medical records and compared in 2 groups. Serum levels of TSH were measured by enzyme-linked immunosorbent assay in all participants.

### Ethical considerations

The study proposal was approved by the ethics committee of Shahid Sadoughi University of Medical Sciences, Yazd, Iran (Code: IR.SSU.MEDICINE.REC.1398.074).

### Statistical analysis

All statistical analyses were performed using the Statistical Package for the Social Sciences (version 16.0, SPSS Inc., Chicago, Illinois, USA). The data were reported as a mean, standard deviation, number, and percentage. The Student *t *test compared the quantitative variables, and to compare the frequency of qualitative data, a Chi-square test was applied. P-values < 0.05 were considered significant.

## 3. Results

In total, 430 medical records of women referred to the Yazd Reproductive Sciences Institute from December 2018-March 2021 were reviewed in 2 groups: case group (unexplained infertility) and control group (male factor infertility). 80 participants in the case group and 50 from the control group were excluded because of endocrine disorders such as polycystic ovary syndrome, hypo- or hyperthyroidism, incomplete medical record information, and other exclusion criteria. Finally, the data of 300 infertile women in 2 groups (n = 150/each) were analyzed.

The mean ages of the women in the case and control groups were 31.50 ± 3.24 and 31.55 ± 3.79 yr, respectively (p = 0.909). Also, the women in the case and control groups had a similar mean BMI (23.48 ± 1.82 and 23.20 ± 2.01 kg/m^2^, respectively) (p = 0.204). No significant differences were observed between the 2 groups regarding demographic characteristics, including the range of age and BMI (Table I).

**Figure 1:**
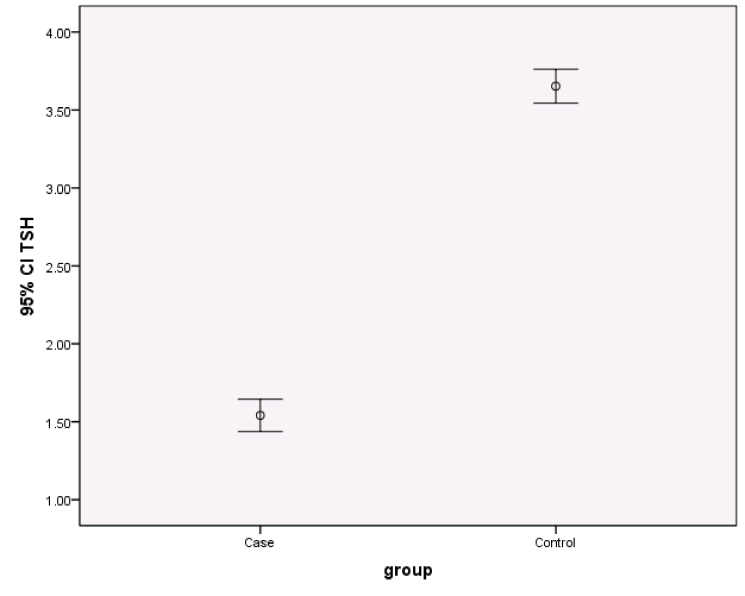
The TSH range in the 2 study groups (CI: 95%).

Our findings showed that TSH levels were significantly higher in women with unexplained infertility than the controls (3.65 ± 0.62 vs. 1.54 ± 0.64, respectively, p < 0.001). The TSH range in the 2 groups is shown in figure 1.

Also, we evaluated serum TSH levels in terms of age and BMI between the 2 groups. It was shown that serum TSH levels were not significantly associated with increasing age and BMI (Table II).

**Table 1:** Comparison of demographic characteristics in 2 study groups (n = 150)


**Variables**		**Case group**	**Control group**	**P-value**
**Age (yr)**				
	**24–30**	61 (40.7)	54 (36)	
	**31–39**	89 (59.3)	96 (64)	0.40
**BMI (kg/m^2^)**				
	**19.5–24.9**	110 (73.3)	118 (78.7)	
	**≥ 25**	40 (26.7)	32 (21.3)	0.27
Data presented as n (%), Chi-square test, BMI: Body mass index				

**Table 2:** Comparison of the TSH levels according to age and BMI in 2 study groups (n = 150/each)


	**Age (yr)**				**BMI (kg/m^2^)**			
	**Case group**		**Control group**		**Case group**		**Control group**	
**TSH** (**mIU)**	24–30	31–39	24–30	31–39	19.5–24.9	≥ 25	19.5–24.9l	≥ 25
	** < 2.5**	3 (4.9)	6 (6.7)	49 (90.7)	84 (87.5)	8 (7.3)	1 (2.5)	107 (90.7)	26 (81.3)
	** ≥ 2.5**	58 (95.1)	83 (93.3)	5 (9.3)	12 (12.5)	102 (92.7)	39 (97.5)	11 (9.3)	6 (18.8)
**P-value**	0.64		0.54		0.27		0.13	
Data presented as n (%), Chi-square test, BMI: Body mass index, TSH: Thyroid-stimulating hormone								

## 4. Discussion

The present study compared the serum TSH levels of 150 women with unexplained infertility and 150 healthy women (with male factor infertility). The findings showed that serum TSH levels were significantly higher in women with unexplained infertility than in healthy women. Also, serum TSH levels had no significant relationship with the women's age and BMI.

Other studies have found associations between higher TSH levels and unexplained infertility in euthyroid women. For example, a study, conducted in Pakistan, reported higher TSH levels in women with unexplained infertility than in controls (1, 3). A study by Sowiński et al. similarly found that mean serum TSH levels were significantly higher in infertile women versus fertile women (0.84 ± 1.96, and 0.38 ± 0.52 mU/ml, respectively) (7). Another study also found higher serum TSH levels among infertile women (8).

Given that all women in the present study had TSH in the normal range, it seems that poor changes in the TSH levels in the normal range may also be an important factor in women with unexplained infertility. However, there is a lot of controversy in studies about the high level of TSH. A study by the American Society for Nutrition reported a mean TSH level of 1.5 mIU/L and an upper limit of 6.10 mIU/L in women and girls without thyroid disease. According to the National Academy of Clinical Biochemistry, 95% of people who have no evidence of thyroid disease have TSH levels below 2.5 mIU/L. Therefore, the upper limit of normal TSH in this study was considered 2.5 mIU/L. Because TSH levels above 2.5 mIU/L are associated with complications such as an increased risk of miscarriage (1).

However, the results of some studies are inconsistent with our findings. In a retrospective cohort study, researchers compared the pregnancy outcomes in 156 euthyroid infertile women who underwent intrauterine insemination due to unexplained infertility in 2 groups: serum TSH levels of 0.5–2.49 mIU/L and serum TSH levels of 2.5–4.5 mIU/L. They concluded that no statistically significant differences were observed between the groups regarding live birth rate, clinical pregnancy, and abortion rate. Therefore, it was concluded that TSH levels 0.5–4.5 mU/L before pregnancy had no significant effects on IUI outcomes of euthyroid women with negative thyroid antibodies (9). Moreover, Diamond et al. could not detect a significant effect of serum TSH levels in women with unexplained infertility (6). Another study concluded that TSH levels were not statistically significant between fertile and infertile women (10). Other researchers found no statistically significant difference between high TSH levels and pregnancy rate (11). A recent study in 2023 found that unexplained infertile women with high-normal TSH levels treated with levothyroxine had higher pregnancy rates but lower live birth rates compared to untreated women (12).

The effect of TSH levels before pregnancy on assisted reproductive technology (ART) outcomes is under discussion. While several studies were unable to show the relationship between TSH levels and pregnancy outcomes in ART (13–15), the relationship between thyroid dysfunctions and the pathogenesis of infertility were discussed in a few studies. Some studies have shown that thyroid autoimmunity affects infertility through human gonadotropin receptors and other placental antigens and finally reduces TSH levels in infertile women (16).

Our study was found to be different from the mentioned studies since only women with unexplained infertility were included to have a more homogeneous study population and to avoid the confused effects of infertility. Some studies have shown an association between thyroid antibodies and pregnancy loss (17, 18), in this study, women with hypo/hyperthyroidism and those with positive thyroid antibodies were excluded. Eliminating this factor can help validate our findings.

### Limitations

This study had the following limitations: 1) it was a single-center study, and 2) lack of antithyroid peroxidase antibody (anti-TPO) measurement in participants. Therefore, to detect more accurate effect of TSH level < 2.5 mIU/L in female infertility, a 3–6 months treatment period is suggested for future studies, in order to reduce TSH below 2.5 mIU/L in these women on the ART outcomes and the effect of anti-TPO.

## 5. Conclusion

TSH levels were higher in women with unexplained infertility compared to the control group, so studying TSH levels in women referring to the reproductive centers to use the methods of assisted reproduction is essential.

##  Data availability

Data supporting the findings of this study are available upon reasonable request from the corresponding author.

##  Author contributions

RA, AA, and MJ designed and conducted the research. RA and AGhA monitored, evaluated, and analyzed the results of the study. Further, RA, AA, AGhA, and MJ drafted the manuscript and reviewed it. All authors approved the final manuscript and take responsibility for the integrity of the data.

##  Conflict of Interest

The authors declare that there is no conflict of interest.
